# The Effects of the Deacetylation of Chitin Nanowhiskers on the Performance of PCL/PLA Bio-Nanocomposites

**DOI:** 10.3390/polym15143071

**Published:** 2023-07-17

**Authors:** Ivan Kelnar, Ludmila Kaprálková, Pavel Němeček, Jiří Dybal, Rasha M. Abdel-Rahman, Michaela Vyroubalová, Martina Nevoralová, A. M. Abdel-Mohsen

**Affiliations:** Institute of Macromolecular Chemistry, Czech Academy of Sciences, 162 00 Prague, Czech Republic

**Keywords:** poly(lactic acid), poly(ε-caprolactone), chitin nanowhiskers, deacetylation, dynamic asymmetry

## Abstract

The multiple roles of organic nanofillers in biodegradable nanocomposites (NC) with a blend-based matrix is not yet fully understood. This work highlights combination of reinforcing and structure-directing effects of chitin nanowhiskers (CNW) with different degrees of deacetylation (DA), i.e., content of primary or secondary amines on their surface, in the nanocomposite with the PCL/PLA 1:1 matrix. Of importance is the fact that aminolysis with CNW leading to chain scission of both polyesters, especially of PLA, is practically independent of DA. DA also does not influence thermal stability. At the same time, the more marked chain scission/CNW grafting for PLA in comparison to PCL, causing changes in rheological parameters of components and related structural alterations, has crucial effects on mechanical properties in systems with a bicontinuous structure. Favourable combinations of multiple effects of CNW leads to enhanced mechanical performance at low 1% content only, whereas negative effects of structural changes, particularly of changed continuity, may eliminate the reinforcing effects of CNW at higher contents. The explanation of both synergistic and antagonistic effects of structures formed is based on the correspondence of experimental results with respective basic model calculations.

## 1. Introduction

In spite of the well accepted fact of the higher effectivity of various nanofillers (NF) in multicomponent polymer systems in comparison to single-matrix nanocomposites, the research has so far been mostly focused on systems with inorganic/carbon-based NF [1,2,3] but rarely on those with organic nanoparticles [4,5,6,7]. In this area, an important class is represented by systems based on natural polymers, like cellulose [4], chitin [8] and silk fibroin [7]. Their application in biodegradable and biocompatible polymer-based matrices especially only allows for the preparation of nanocomposites (NC) without any compromising of their performance. Chitin nanowhiskers (CNWs) are prepared from chitin, the second most abundant biopolymer, which is rigid crystalline nanofibre with Young’s modulus of 40–80 GPa. It is composed of glucosamine and N-acetylglucosamine repeating units bearing reactive groups, i.e., amines. Thus, chitin has higher potential for chemical modifications than cellulose. Chitin has three polymorphs, α, β and γ, which have different degrees of molecular packing. CNW may be produced in a relatively wide range of length, diameter, charge density, type of charge and crystallinity via diverse top-down procedures [9,10,11,12] from different raw materials of animal or fungal origin. In the case of NC, CNWs of animal origin have so far been of higher interest. In the case of biodegradable nanocomposites, CNWs are mostly applied in poly(ε-caprolactone) (PCL) [13,14], poly(3-hydroxybutyrate-co-3-hydroxyvalerate) (PHVB) [15,16], poly(lactic acid) (PLA) [17,18] and plasticized PLA [19] matrices. Of crucial importance is fair dispersion; solution-based techniques and melt mixing, including water-assisted extrusion [20], are mostly applied. Dispergation and parameters of interface can be improved by grafting of polymer chains [16,21]. The only application of CNWs in a blend matrix was reported for PLA/PHBV [22]. Generally, the effect of NF is more complex in partially miscible mixtures with phase separation [23], but affecting of dynamic phase behaviour in immiscible systems combined with formation of complex morphologies may also lead to synergistic effects, causing a simultaneous increase in stiffness, strength and toughness [24,25]. Of importance is also the NF affecting of dynamic asymmetry enabling control of continuity [26]. In the case of immiscible blends of biodegradable polyesters, combination of rigid brittle PLA/low modulus tough PCL [27,28,29] can provide a material with a broad range of mechanical properties. Here, successful application of inorganic NF, such as MMT [30] TiO_2_ [31] and carbon-based NF [32], which can also eliminate low compatibility between PCL and PLA, was reported. We have found important effect of graphite nanoplatelets in dependence on PCL/PLA blend ratio [32]; importance of NF localization controlled by modification and functionalization was demonstrated in the PCL/PLA/GO system [33]. In microfibrillar composites based on the PCL/PLA blend, NF only allows their effective preparation [34]. A few studies using organic NF in PCL/PLA deal mostly with cellulose nanocrystals (CNC) [19], including PCL- and PLA-grafted CNC [6,7]. Recently, we have revealed the role of CNC and dopamine-coated CNC in PCL/PLA of different blend ratios [35]. In the case of CNW application in biodegradable polyesters, aminolysis must also be considered [20,36]. To the best of our knowledge, this effect on performance of blends of various polyesters has not been published. Therefore, the present study is devoted to the effect of the degree of surface deacetylation (DA) of CNW and mixing protocol, and also to controlling CNW localization on the structure and properties of the PCL/PLA-matrix bionanocomposite.

## 2. Experiment

### 2.1. Materials

Poly(lactic acid) (PLA) Ingeo 2002D (Nature Works, Plymouth, MN, USA) with a D-isomer content of 4.3%, Mw 2.53 × 10^5^ g·mol^−1^, melt-flow rate of 6 g/10 min (190 °C/2.16 kg), and density of 1.24 g·cm^−3^. Poly(ε-caprolactone (PCL) CAPA 6800 (Perstorp, Malmö, Sweden) Mn 8·10^4^ g·mol^−1^, melt-flow rate 3 g/10 min (160 °C/2.16 kg) and density 1.145 g·cm^−3^. Dimethyl formamide (DMF) was purchased from Sigma Aldrich (Saint Louis, MO, USA). Chitin shrimp shell was purchased from Sigma Aldrich (Darmstadt, Germany). Hydrochloric acid, acetic acid and ethyl alcohol were purchased from Penta chemical company (Prague, Czech Republic).

#### Preparation of Chitin Nanowhiskers

Pure chitin was extracted from shrimp shells according to our previous work [37]. In brief, the extraction consisted of three main steps: demineralization (DM), deproteinization (DP) and deacetylation (DA). The DM step was carried out using aqueous hydrochloric acid at ambient temperature (25 ± 2 °C) for 24 h. In the DP step, aqueous NaOH was used to remove the non-bounded materials like proteins, dyes, lipids and pigments. The crude chitin was stirred in 5% of NaOH at 90 °C for 24 h. The product was filtered off, washed and dried at 60 °C for 12 h.

The partial deacetylation of chitin was achieved by refluxing the chitin with (50% *w*/*v*) of sodium hydroxide solution at 90 °C with solid-to-solvent ratio (1/30 *w*/*w*) for 5 h. Partially deacetylated chitin was collected, washed with Milli-Q water until neutral pH was achieved, rinsed with ethanol, vacuum-filtered and dried at 60 °C for 12 h to remove moisture. The degree of decatylation was 30%, confirmed FTIR spectroscopy in combination with XRD (Figure S1).

Chitin (CNW_2_) and partially deacetylated chitin nanowhiskers (CNW_1_) suspensions were prepared by acid hydrolysis using different concentrations of hydrochloric acid (5 M) at 90 °C for a variable time (1 to 6 h) under mechanical stirring_ENREF_27. The solid–liquid ratio of the chitin suspension was (1/100). After the acid hydrolysis, the suspension was diluted with Milli-Q water and centrifuged at 4000 rpm. The process was repeated five times until a pH of approximately 4 was achieved. The suspension was transferred to a cellulose dialysis bag (molecular cut 12–14 kDa) and dialyzed in Milli-Q water until the chitin nanocrystals suspension was sufficiently neutral (pH = 6–6.5).

### 2.2. Nanocomposite Preparation

To prepare 10% masterbatches, water suspensions of CNW were converted to DMF and ultrasonication-dispersed. After addition of PLA or PCL (Polymer/CNC ratio was 9/1 *w*/*w*) and mixing at 85–90 °C for 2.5 h, the solvent was evaporated at 50 °C for 24 h.

The nanocomposites were prepared by melt-mixing in Haake Mini CTW at 140 °C (PCL) and 190 °C (PLA and blend) for 5 min at 45 rpm. CNW_1_ and CNW_2_ were added as 10% masterbatch in PCL or PLA. Two sets of samples differing in addition of CNW via PCL- or PLA-masterbatch with 50/50 PLA/PCL weight ratios were prepared. Composition of samples is shown in Table S1. The masterbatch and polymers were combined to achieve 2% CNW1 or CNW2 content in the nanocomposites. In the CNW-free samples, PLA or PCL analogously treated with dimethyl formamide were applied. Subsequently, a film of ~0.2 mm thickness was prepared in a laboratory press (140 °C for 5 min).

### 2.3. DFT Modelling of Stabilization Energy between Constituents

For closer insight into the interactions of CNW_1_ and deacetylated CNW_2_ with polymer components, we carried out model quantum-chemical calculations of interactions between the structural units of CNW, i.e., chitin and chitosan, and the respective polymer chains. Hydrogen bonding effects were studied at the DFT level of theory with the B3LYP exchange correlation functional in combination with the semi-empirical dispersion correction GD3BJ [38]. Calculations with the 6–31 ± G (d,p) basis set were performed with the Gaussian 16 program package [39]. The fully optimized geometries represent the true energy minima on the potential energy surface. Here, no imaginary frequencies were obtained with normal mode calculations. Moreover, in the calculations of the stabilization energies of the hydrogen-bonded complexes (the difference between the energy of two interacting structural units and the complex), the Boys and Bernardi counterpoise correction was applied in order to consider the basis set superposition error [40].

### 2.4. Characterization of Blends Structure

The structure was examined using scanning electron microscopy (SEM) with a Maia microscope (FEI, Brno, Czech Republic). The injection-moulded specimens broken under liquid nitrogen were etched in 20% NaOH for 20 min to remove the PLA component in the PCL matrix and bicontinuous samples; the PLA matrix samples were etched with THF vapour at 45 °C for 4 min to “visualize” PCL inclusions [41].

### 2.5. Testing

Tensile tests were carried out using an Instron 5800 (Instron, High Wycombe, UK) apparatus on dog bone samples cut from the films at 22 °C and crosshead speed of 1 mm/min (ISO 527-2) [42]. At least eight specimens were tested for each sample. Young’s modulus (*E*), stress at break (*σ*) and elongation at break (*ε*_b_) were evaluated.

Tensile impact strength, *a*_t_, was measured on unnotched injection-moulded samples using CEAST Resil impact junior hammer (CEAST S.p.A., Torino, Italy) with an energy of 4 J. The reported values are the averages of ten individual measurements.

The DSC analysis was performed with a TA Instruments Q2000 DSC apparatus (New Castle, DE, USA). The measurements were carried out in a heating–cooling–heating regime between 0 °C and 200 °C at a constant heating and cooling rate of 10 °C/min. The values of 139.5 J/g and 93.7 J/g were used as the melting enthalpies of 100% crystalline PCL and PLA, respectively. The final crystallinity values were calculated in relation to the real weight fraction in the sample.

The rheological characterization was carried out using an ARES apparatus (Rheometric Scientific, Piscataway, NJ, USA) with the parallel-plate geometry at 170 °C using an oscillatory shear deformation within the frequency range of 0.1–100 rad/s. The amplitude of oscillation was 1%.

Dynamic mechanical analysis (DMA) was performed in single-cantilever mode using a DMA DX04 T apparatus (RMI, Pardubice, Czech Republic) at 1 Hz and heating rate of 1 °C/min from −120 to 150 °C.

## 3. Results and Discussion

### 3.1. Effect of CNW Surface Deacetylation on Aminolysis of Polyester Chains

**Figure 1:**
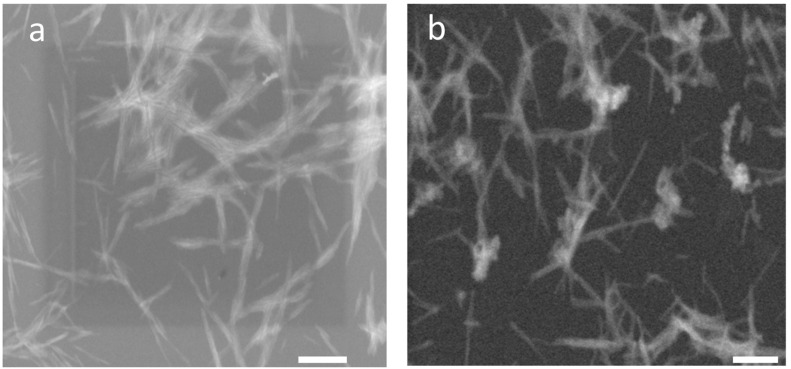
STEM images of CNW with different degree of deacetylation: (**a**) CNW_1_ with 30% DA, (**b**) CNW_2_ with 5% DA.

**Figure 2:**
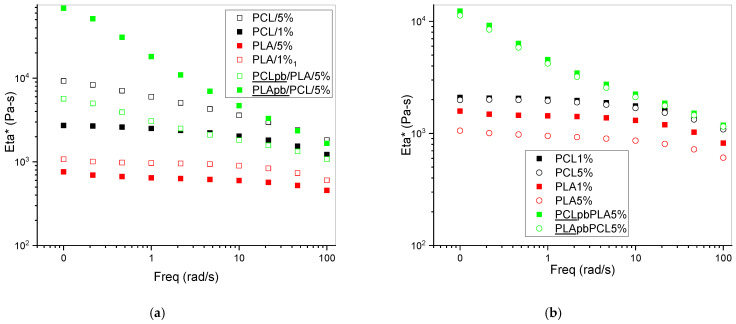
(**a**) Viscosity of PCL, PLA and PCL/PLA 50/50 containing CNW_1_ (~30% deacetylation). (**b**) Viscosity of PCL, PLA and PCL/PLA 50/50 containing CNW_2_ (<5% deacetylation). Eta*—complex viscosity.

Figure 1 and Figure S1 show similar aspect ratios for both CNWs together with FTIR and XRD, which is confirmation of different DAs. As a result, the differences in rheological behaviour (Figure 2) are caused most of all by expected aminolysis of polyesters by primary or secondary amines on the surface of the CNW to form secondary and tertiary amides accompanied by expelling of OH-containing chain fragments (Scheme S1). From the results in Figure 2a,b it follows that viscosity of PLA containing 5% of either CNW1 or CNW2 is lower than with 1% NF content. This comparable marked drop in viscosity indicates high degree of scission of esters accompanied by attachment of CNW (Scheme S1) and thus comparable reactivity of CNW_1_ containing primary amines (30% deacetylation) and CNW_2_ with dominating secondary amine. At the same time, in agreement with lower ester bonds content in PCL, its scission is less extensive in comparison to PLA. Figure 2a shows higher viscosity of PCL with 5% CNW_1_ against 1% content and less marked drop for 5% CNW_2_ against 1% in comparison to PLA (Figure 2b).

The surprising more marked scission of the “less reactive” PCL with CNW_2_ containing more secondary amines (nondeacetylated amides), which may correspond to different dispergation (and dimensions) and thus interfacial area, is worth further study. Unfortunately, in the case of evaluation of the extent of the reaction (Scheme S1), e.g., by highlighting the content of newly formed amide bonds between CNW and polyester chain, in addition of low resolution of FTIR for analysis of composite samples, we were limited also by a low effectivity of separation of the grafted CNW from the matrix. In spite of this, preliminary results (Figure S2) with CNWs separated as filter cake confirm different extent of grafting of PLA and PCL to CNWs. More pronounced shear thinning for the PCL/PLA nanocomposite in comparison to the single-matrix nanocomposite indicates that shear thinning caused by CNW orientation with increasing shear rate is accompanied by some ordering of bicontinuous structure [43] as well. Moreover, due to similar viscosity (deformability) of PCL and PLA, we consider that the possible reduction in the continuous phase content at the expense of its localization as inclusions in the threads of the second polymer [44] has limited impact on rheological performance.

### 3.2. Effect of CNW Functionality and Localization on Structure

Taking into account the above-shown significant effects of both CNW on PLA viscosity reduction and somewhat more marked drop for PCL with CNW_2_, we can consider a relatively significant affecting of the viscosity ratio [45], especially at higher CNW content. Moreover, expected different extent of aminolysis of PCL by CNW_1_ and CNW_2_ (Figure 2) leads to different content of PCL/CNW adducts with compatibilising ability. These effects, also affected by CNW localization, undoubtedly represent the main contributions to differences in structure of blends and related NC.

Due to the poor “visibility” of CNW in polymer systems [35], estimation of its localization is based on the dominancy of kinetic factors [45], like targeted pre-blending, over thermodynamic ones [46]. The reason is the limited migration of the relatively large nanoparticles during short-time melt mixing. At the same time, localization of a CNW in a thermodynamically less favourable phase may also lead to its increased presence at the interface, which may have a significant impact on its properties. Due to low reliability of wetting coefficients evaluation [46], we have applied a DFT calculation of interaction energy between basic building blocks of respective components. Scheme S2 shows higher interaction energy between both CNW and PLA in comparison to PCL; due to their similar viscosity, we expect for PLA/CNW pre-blend (PLApb) application localization of CNW predominantly in this phase, whereas for PCL/CNW pre-blend some migration to PLA phase can also be considered.

**Figure 3:**
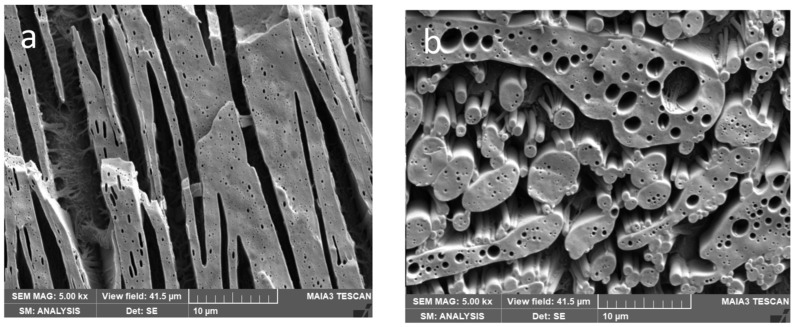
Structure of neat blends: (**a**) DMF-treated PCL(PCL_DMF_)/PLA, (**b**) DMF-treated PLA(PLA_DMF_)/PCL.

Taking into account certain negative effects of DMF on the polyesters applied [35], CNW-free blends were also prepared using DMF-treated (analogously to masterbatch preparation) components. The different structures for PLA_DMF_/PCL and PCL_DMF_/PLA (Figure 3), especially lower continuity and higher amounts of the PLA subinclusions inside the PCL phase for PLA_DMF_ confirm the altering of rheological parameters and, thus, also of dynamic asymmetry [26] by DMF.

**Figure 4:**
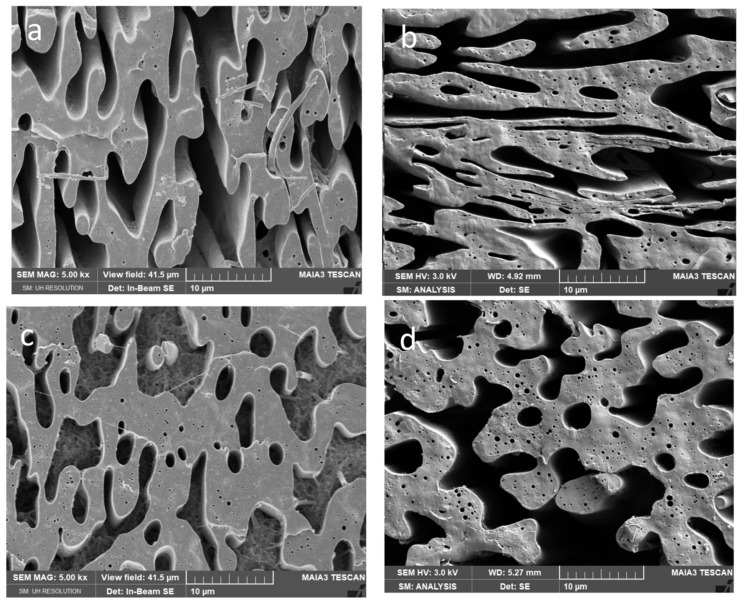
SEM images of 50/50PCL/PLA with 1% CNW: (**a**) PCLpb/PLA/CNW_1_, (**b**) PCLpb/PLA/CNW_2_, (**c**) PLApb/PCL/CNW_1_, (**d**) PLApb/PCL/CNW_2_.

**Figure 5:**
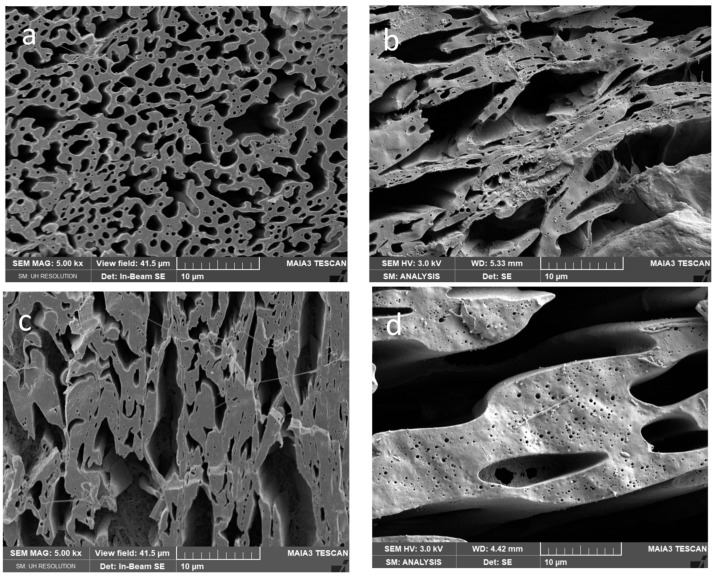

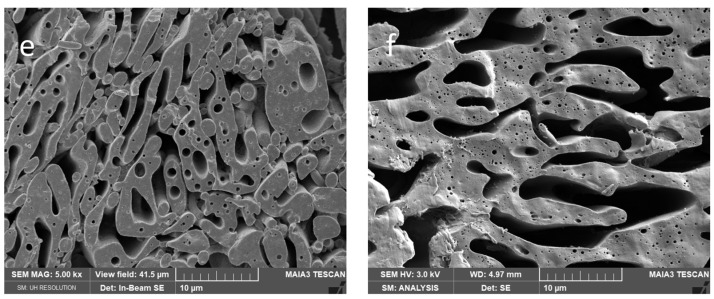
SEM images of 50/50PCL/PLA with 2% CNW: (**a**) PCLpb/PLA/CNW_1_, (**b**) PCLpb/PLA/CNW_2_, (**c**) PLApb/PCL/CNW_1_, (**d**) PLApb/PCL/CNW_2_, (**e**) PCLpb/PLApb/CNW_1_, (**f**) PCLpb/PLApb/CNW_2_.

**Figure 6:**
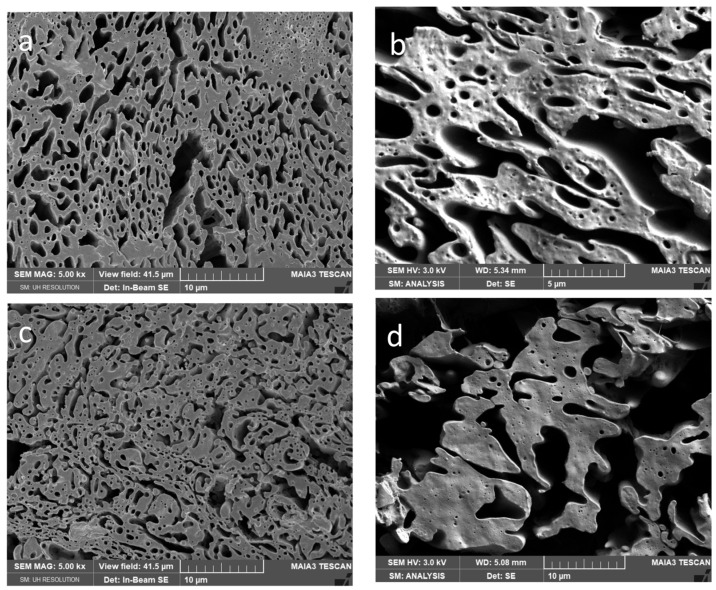
SEM images of 50/50PCL/PLA with 5% CNW: (**a**) PCLpb/PLA/CNW_1_, (**b**) PCLpb/PLA/CNW_2_, (**c**) PLApb/PCL/CNW_1_, (**d**) PLApb/PCL/CNW_2_.

From Figure 4, Figure 5 and Figure 6 it follows that presence of CNW in PCL or PLA leads to more marked changes in the “relative” continuity (of one phase at the expense of the other) and roughness of the threads. These structural changes are more pronounced with increasing CNW content. Due to a more marked decrease in the viscosity of PLA by both CNW_1_ and CNW_2_ (Figure 2) and the mentioned dominant presence of CNW in this phase, the application of PLA pre-blends leads to lower continuity of this phase (in contrast to nonreactive CNC [35]). Further, important effects on structure originate from CNW bonded to polymers (Scheme S1) and CNW localized at the interface.

From Figure 6 showing systems with 5% CNW, it is especially clear that increasing of CNW content leads to most marked changes in continuity, and an increase in the roughness of threads also accompanied by a higher content of inclusions with varied size and shape. Generally, in all systems we mostly suppose the presence of spheres, but the presence of elongated fibrous inclusions (including tiny threads) inside threads cannot be excluded. They cannot be practically observed by SEM and the etching techniques applied; some indications show fracture surfaces (Figure S3) but brittle PLA is probably not pulled out sufficiently. We consider that the presence of PLA fibres is also indirectly indicated by the unexpected high stiffness of some composites (see below).

From SEM images, it further follows that the difference between structures of nanocomposites containing CNW_1_ and CNW_2_ are relatively low, which seems to be in agreement with moderate differences in the components viscosity (see Figure 2).

### 3.3. Mechanical Properties of Blends and Nanocomposites

Table 1 shows similar reinforcing effects of CNW_1_ and CNW_2_ for both PCL and PLA components corresponding to their similar aspect ratio AR (Figure 1) and the extent of the reaction, especially with PLA obvious from rheology (Figure 2). We can see a relatively fair correspondence to increasing CNW content.

**Figure 7:**
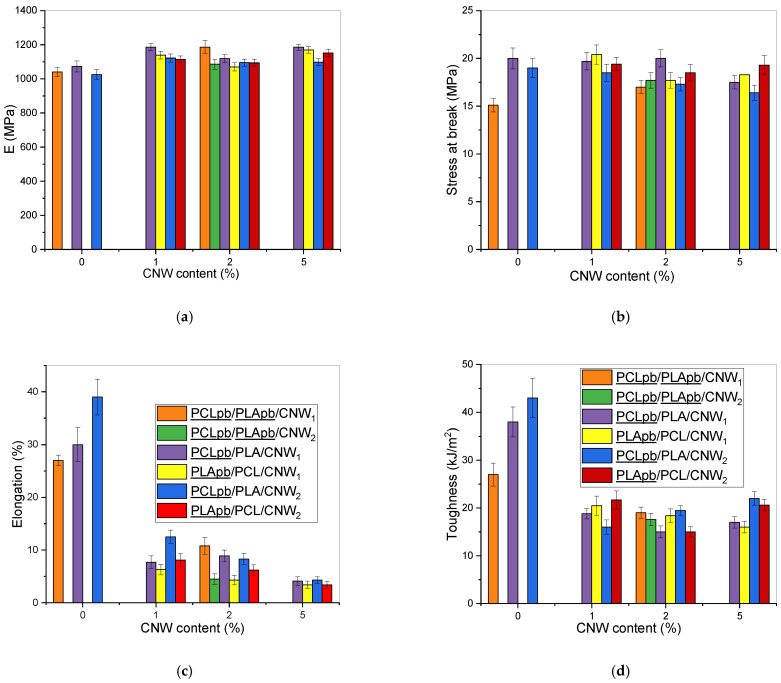
Mechanical properties in dependence on CNW content and mixing protocol (**a**) Young’s modulus E; (**b**) stress at break; (**c**) strain at break; (**d**) toughness. Legend to all graphs are shown in (**c**,**d**), e.g., PCL/PLApb/CNW_2_ means system prepared using PLA pre-blend; PCLbp/PLApb without CNW application means DMF treated components.

In the case of NCs with the PCL/PLA matrix, Figure 7a–d shows that mechanical properties are mostly better for the PCL pre-blend with both CNW_1_ and CNW_2_. The reason is a decrease in the continuity of the PLA phase (see above and Figure 4, Figure 5 and Figure 6). The performance of a reactive CNW is in contrast with CNC, where the PLA pre-blend leads to higher continuity [38] of this phase and related better properties. At the same time, we can practically exclude the contribution of CNW and structural changes in crystallinity; in addition to low differences in crystallinity of all systems (see Section 3.6), a specific feature of PCL and PLA is low differences between amorphous and crystalline phase properties [30]. Therefore, we can only consider the practically undetectable affecting of interface by changes in crystallinity [47] in this area, which may be relatively low as well, due to the above facts.

Of interest is the fact that the best effectivity of CNW was found only in nanocomposites containing 1% CNW, with practically no increase in properties with its higher content. This is in contrast with a single NC (Table 1). To explain this peculiarity, our experience [27,38] and basic model calculations [48,49,50] describing the effect of structure type on modulus (Table S2) indicate that CNW- (and DMF-) induced changes in structure, including subinclusions of content and shape, may lead to a significant affecting of mechanical properties which may reduce or even eliminate reinforcing effect of CNW, e.g., a relatively high *E* of neat blend and NC with 1% CNW_1_ using PCL pre-blend (in comparison to *E* from Davies’ model [48] for “pure” bicontinuous structure, see Table S2) can be due to formation of PLA fibres instead of inclusions in PCL. From Table S2, it follows that, if the PCL phase contains 10% of PLA fibres with aspect ratio 10 instead of spheres, the modulus is ~10% higher.

A positive effect on *E* comes also from the presence of the PCL subinclusions in PLA at the expense of the continuous PCL phase, whereas the analogous presence of the PLA inclusions in the PCL has an opposite effect (see Table S2). Table S2 further shows that reinforcement of either PCL or PLA by preferred CNW localization has a negligible effect on the composite parameters. Finally, of some importance may be practically non-detectable changes (decreases) in the modulus of the interface, e.g., a lower content of spherulites in this area [47]; however, this effect is undoubtedly limited, as mentioned above.

In the case of all other parameters (Figure 7b–d), we can see both a higher decrease with CNW content and more significant differences between samples with different mixing protocols and CNW types. This indicates an even higher impact of a less favourable rougher structure, changed parameters of continuous threads and the interface on properties accompanied with a higher extent of deformation. This is confirmed by most marked deviations in the case of elongation (Figure 7c). Low correspondence or even dissimilar trends between elongation and toughness (Figure 7d) are in agreement with the performance of viscoelastic materials at different straining rates, especially in combination with structural changes, also influencing, e.g., energy-absorbing micro-deformations [51]. To conclude, it is clear that CNW-induced structural changes can eliminate its reinforcement; the differences between CNWs with high and low DAs are relatively low due to similar effect on components aminolysis. The presence of obvious significant antagonistic effects with high CNW content is especially worth more detailed study.

### 3.4. Dynamic Mechanical Analysis

Table 1 shows a slight increase in *T*_g_ for both a single PLA and a PCL nanocomposite with CNW_1_, whereas a decrease mostly occurs for CNW_2_, in all cases with low correspondence to the CNW content. This indicates engagement of more effects than mere immobilization of polymer chains and corresponds to the still not fully understood effects of NFs on polymers [52]. Similar results were observed in systems with MMT, HNT and CNC. For instance, 2% CNC led to slight decrease for PLA and increase for PCL [35].

**Figure 8:**
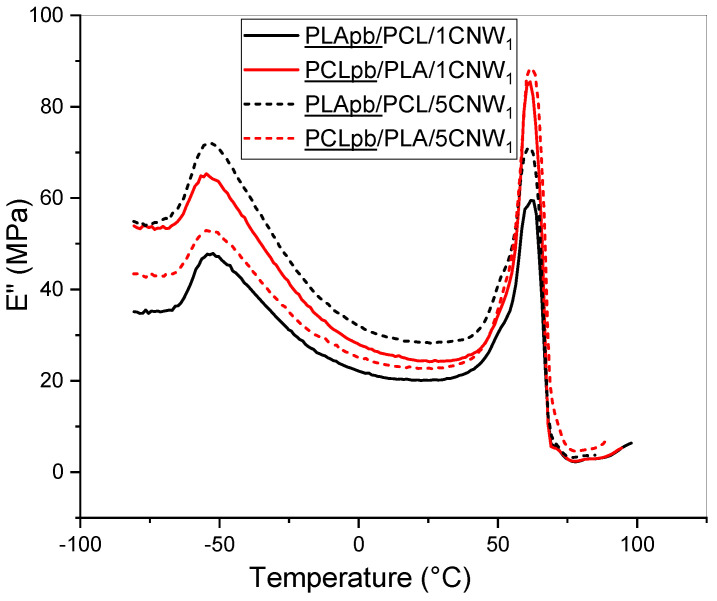
Thermal dependence of loss modulus in dependence on mixing protocol and CNW_1_ content (e.g., PLApb/PCL/1CNW_1_ means NC with 1% CNW_1_ prepared using PLA pre-blend).

Examples of thermal dependence of the loss modulus for the PCL/PLA system with the PCL/CNW_1_ or PLA/CNW_1_ pre-blend are shown in Figure 8; the *T*_g_ of all systems prepared are in Table S1. It is obvious that trends (shifts of *T*_g_) observed for blend-based NC prepared using PCL/CNW or PLA/CNW pre-blend mostly follow those found for a corresponding single-matrix NC, but usually more significant altering of both *T*_g_ values indicates the presence of further effects. Especially in the case of parallel using of the pre-blends of both polymeric components with CNW_1_ or CNW_2_, a certain merging of both *T*_g_s indicates their compatibilising effect. The more “asymmetric” changes in respective PCL/CNW or PLA/CNW pre-blends, also with a higher probability of interfacial localization of CNW, correspond to CNW-induced changes in the structure and interface. A minor decrease in the *T*_g_ of PLA may be also caused by a PCL dissolved in the PLA phase [53]. In particular, formation of a complex hierarchical structure most probably leads to micromechanical transitions [54], which may significantly influence *T*_g_ in oscillatory straining-based measurements. It is obvious that the DMA results correspond to marked structural transformations dependent on NF type, content and localization.

### 3.5. TGA

**Figure 9:**
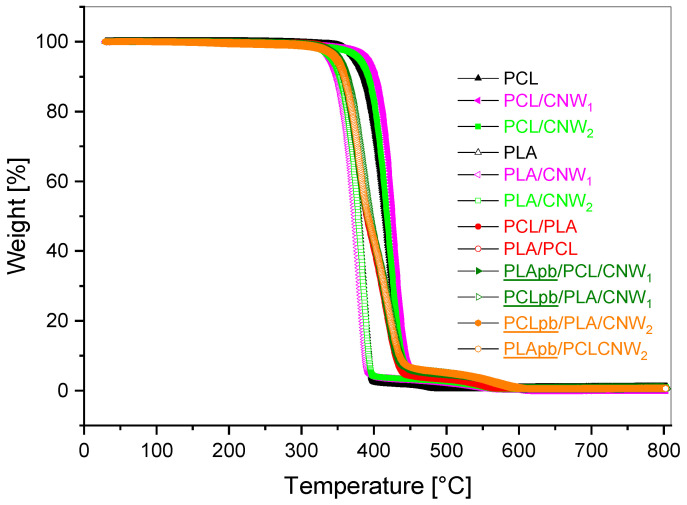
TGA curves for single and blend-based matrix nanocomposites, CNW content 5%.

Figure 9 indicates negligible differences in thermal stability between all systems, i.e., a single NC and blend-based NC with different mixing protocols and CNW content for both CNW_1_ and CNW_2_. Hence, we can conclude that the extent of CNW deacetylation, i.e., different content of primary and secondary amines, has a low impact on thermal stability and, in fact, it also confirms similar ability of both primary and secondary amine at the CNW surface for aminolysis of polyesters.

### 3.6. Effect of CNW on Crystallinity

In the case of a single PCL, the addition of CNW_1_ with a higher deacetylation has little effect on the crystallinity (Table 2); it leads to a slight decrease, whereas the presence of CNW_2_ causes an increase, which is in contrast to the negative effect of CNWs mentioned in the literature [55]. This may consist of different degrees of PCL scission by CNW_1_ and CNW_2_, and also the related content of CNW with attached PCL chains (indicated by rheology, see Section 3.1). With a single PLA, both CNWs cause some reduction in the initial crystallinity accompanied by higher extent of cold crystallization (CC), which is more marked for CNW_2_ in both cases. This behaviour indicates certain hindering of the chain mobility accompanied by a nucleation effect on CC. In particular, a reduction in *T*_cc_ and a small increase in total crystallinity for PLA/CNW are in agreement with results of others [56,57].

In all nanocomposites with the PCL/PLA matrix, the mentioned effects of CNW on (initial) crystallinity of the components, especially PLA, seem to be eliminated. Depending on the mixing protocol, different degrees of decrease in PCL crystallinity and relatively significant differences in CC of PLA occur. More marked suppressing of CC was found for CNW_2_. The only exception is the “symmetrical“ system prepared using a parallel application of both PCL/CNWpb and PLA/CNWpb, where a higher CC was found. All differences are probably a consequence of changed interface parameters; its area and confinement in the case of fine inclusions are inside continuous phases [27]. A positive effect of reduced molecular weight is probably restricted by attachment of CNW to polymer chains (Scheme S1). The relatively small differences in crystallinity are apparently inadequate to mark changes in mechanical properties, as discussed above.

## 4. Conclusions

The results indicate important effects of CNW-induced structure alterations on the mechanical properties of the PCL/PLA-matrix nanocomposites with a bicontinuous structure, which can eliminate reinforcement at a higher nanofiller content. We have also found a marked affecting of the structure by the mixing protocol. The explanation of this performance consists of the negative effect of amines of the CNW surface on molecular weight, especially for PLAs with higher ester group content. According to rheology, aminolysis-scission of PLA chains is practically independent of the extent of deacetylation of CNW and shows only relatively small differences for PCL, probably due to a different area of the interface. This leads to marked altering of dynamic asymmetry, different from analogous systems containing nonreactive cellulose nanocrystals. The structural changes include the degree of continuity of respective phases, their roughness, the content and shape of subinclusions, like spheres, small-branched threads and fibres. As a consequence, favourable combinations of multiple CNW effects leads to improved mechanical performance at 1% only, whereas at higher CNW content, negative effects of structure alterations dominate. This is explained by estimated Young‘s modulus values related to expected structures using corresponding models. An important result is the low effect of the degree of deacetylation of CNW, i.e., the presence of either primary or secondary amine on their surface, on most of the NC parameters evaluated, including thermal stability.

## Figures and Tables

**Table 1:** Effect of CNW_1_ and CNW_2_ on properties of PLA and PCL.

Composition	*E* (MPa)	Stress at Break (MPa)	Strain at Break (%)	Toughness (kJ/m^2^)	*T_g_* (°C)
PLA	2629 ± 261	48.4 ± 4.9	8.1 ± 3.6	12.9 ± 1.8	60.74
PLA/1% CNW_1_	2802 ± 53	32.9 ± 2.1	2.61 ± 0.42	10.8 ± 6.7	61.09
PLA/1% CNW_2_	2779 ± 60	36.6 ± 1.4	2.93 ± 0.50	10.4 ± 5.4	61.03
PLA/2% CNW_1_	2860 ± 70	29.2 ± 3.0	2.66 ± 0.43		
PLA/2% CNW_2_	2843 ± 67	34.8 ± 2.3	3.1 ± 0.50	5.8 ± 3.1	59.22
PLA/5%CNW_1_	2988 ± 62	32.5 ± 2.2	2.15 ± 0.31	23.1 ± 22.8	61.05
PLA/5% CNW_2_	2984 ± 76	36.3 ± 3.1	2.62 ± 0.28	6.57 ± 2.41	59.5
PCL	302 ± 23	27 ± 2.3	505 ± 62	48 ± 3.9	−54.96
PCL/1% CNW_1_	364 ± 18	30.4 ± 2.7	595 ± 49	47.84 ± 4.74	−54.12
PCL/1% CNW_2_	362 ± 22	27.1 ± 4.4	536 ± 71	52.50 ± 7.33	−54.43
PCL/2% CNW_1_	395 ± 30	30.7 ± 1.8	573 ± 11		
PCL/2% CNW_2_	393 ± 9	25.5 ± 3.9	513 ± 57	39.56 ± 7.64	−55.18
PCL/5% CNW_1_	464 ± 36	28.8 ± 3.1	567.9 ± 48.6	48.38 ± 6.62	−53.93
PCL/5% CNW_2_	441 ± 7	20.7 ± 3.3	430 ± 56	29.16 ± 4.45	−54.08

**Table 2:** Effects of CNW on crystallinity of nanocomposites with single and blend-matrix. Cr_PLA_= “total” crystallinity of PLA (initial + cold crystallization); Cr_PLA_-CC_PLA_ = initial crystallinity of PLA.

Composition	CNW (%)	Cr_PCL_ (%)	T_cc_ (°C)	CC_PLA_ (%)	Cr_PLA_ (%)	Cr_PLA_-CC (%)
PCL	-	46.97	-	-	-	-
PCL/CNW_1_	2	45.12	-	-	-	-
PCL/CNW_2_	2	53.24	-	-	-	-
PLA	-	-	112.98	21.64	26.49	4.85
PLA/CNW_1_	2	-	105.73	26.86	29.36	2.5
PLA/CNW_2_	2	-	106.98	28.55	29.45	0.9
PCLpb/PLApb/CNW_1_	2	49.48	99.36	27.04	31.63	4.59
PCLpb/PLApb/CNW_2_	2	48.55	104.99	16.45	23.49	7.04
PCLpb/PLA/CNW_1_	2	41.68	105.88	19.74	27.89	8.15
PCLpb/PLA/CNW_2_	2	38.54	108.09	18.75	25.08	6.34
PCL_DMF_/PLA	-	50.66	116.49	20.9	25.92	5.02
PCL/PLA_DMF_	-	58.91	110.73	24.01	30.62	6.61

## Data Availability

Data are contained within the article.

## Supplementary Materials

The following supporting information can be downloaded at: https://www.mdpi.com/article/10.3390/polym15143071/s1, Table S1. List of samples prepared and T_g_ of polymer components Figure S1: FTIR and XRD of CNWs; Histogram of dimensions; Scheme S1: Aminolysis of polyester chains Figure S2: FTIR of CNW extracted from PCL/PLA/CNW nanocomposite; Scheme S2: DFT evaluation of interaction energy between components; Figure S3: Fracture surface of PCL/PLA/CNW; Table S2: Modulus of PCL/PLA blend in dependence on structure estimated using basic models. References [48,49,50] are cited in the Supplementary Materials.
