# Supplementary material for: The Effects of the Deacetylation of Chitin Nanowhiskers on the Performance of PCL/PLA Bio-Nanocomposites
Source: Polymers (Basel). 2023 Jul 17;15(14):3071. doi: 10.3390/polym15143071 (PMC10384066; doi:10.3390/polym15143071)
Supplement: Supplementary file 1 [file polymers-15-03071-s001.zip › polymers-2507636-supplementary.pdf]

polymers

MDPI

Supplementary Materials

The Effects of the Deacetylation of Chitin Nanowhiskers on the Performance of PCL/PLA Bio-Nanocomposites

Ivan Kelnar *, Ludmila Kaprálková, Pavel Němeček, Jiří Dybal, Rasha M. Abdel-Rahman, Michaela Vyroubalová, Martina Nevoralová and A. M. Abdel-Mohsen

Institute of Macromolecular Chemistry, Czech Academy of Sciences, 162 00 Prague, Czech Republic
* Correspondence: kelnar@imc.cas.cz

Table S1. List of samples prepared and T_g of polymer components: PCL/PLA 50/50 matrix nanocomposite in dependence on mixing protocol, CNW type and content (pb = pre-blend), e.g. PLApb/PCL/CNW_1 means NC with CNW_1 prepared using PLA/CNW_1 pre-blend.

Mixing protocol	CNW content [%]	*Tg* of PCL [°C]	*Tg* of PLA [°C]
PLA_{DMF}/PCL_{DMF}	-	-54.68	60.52
PLA_{DMF}/PCL	-	-53,21	*60,95*
PLA/PCL_{DMF}	-		
PLApb/PCL/CNW_1	1	-53,51	62,31
PLApb/PCL/CNW_2	1	-53.99	+60.35
PCLpb/PLA/CNW_1	1	-54,70	61,03
PCLpb/PLA/CNW_2	1	-54.78	60.00
PCLpb/PLA pb/CNW_1	2	-53,70	60,27
PCLpb/PLA pb/CNW_2	2	-53.34	60.20
PCLpb/PLA/CNW_1	2	-54.64	61.50
PCLpb/PLA/CNW_2	2	-52.93	61.26
PLApb/PCL/CNW_1	2	-52,94	61,75
PLApb/PCL/CNW_2	2	-52.68	60.05
PLApb/PCL/CNW_1	5	-53.73	61.32
PLApb/PCL/CNW_2	5	-54.24	56.55
PCLpb/PLA/CNW_1	5	-53.95	61.85
PCLpb/PLA/CNW_2	5	-51.70	60.71

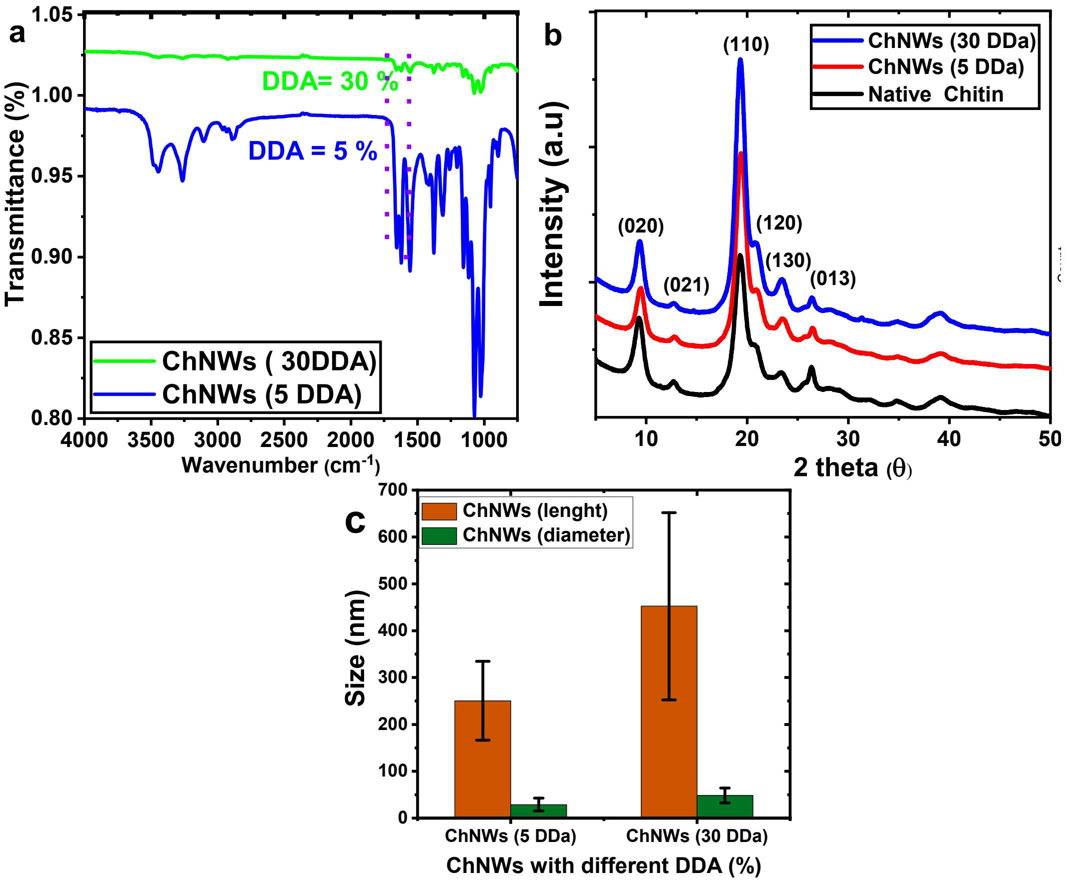

Figure S1. FTIR of CNWs (a); XRD of CNWs (b); Histogram of dimensions of CNWs (c).

Results and disscusion

FTIR spectra show the effect of treatment conditions on the degree of deacetylation of chitin. Fig. **S1a** shows the FTIR of chitin nanocrystals with different DDA (5; 30 %). In α-chitin, two absorption peaks observed at 1655 and 1622 cm^{-1} are related to α-chitin phase. When the deacetylation of chitin occurs, the absorption band assigned to amide II decreases, while the increase in intensity of the amide I band confirms the formation of amino groups. However, in our case, only low DDA shows (30 %) the peak at 1557 cm^{-1} stronger than the peak at 1655 cm^{-1}. Figure **S1b** shows the XRD of native chitin and chitin nanocrytals with different DDA. All native chitin and partially deacetylated chitin nanowhiskers exhibit six diffraction peaks at 2θ = 9.4°, 12.9°, 19.3°, 20.8°, 23.5°, and 26.5 ° indexed as (020), (021), (110), (120), (130), and (013), respectively (Fig. S1b); this suggests a crystalline structure of the α-chitin which fits with the data obtained from FTIR (**Fig. S1a)**. **Figure S1c** shows the effect of chitin treatment on the dimension of NCs. At low DDA (5 %), the length and diameter of ChNCs are slightly smaller compared to ChNCs at high DDA degrees (30 %). This might be due to the small aggregation of NCs at a high free amino group content.

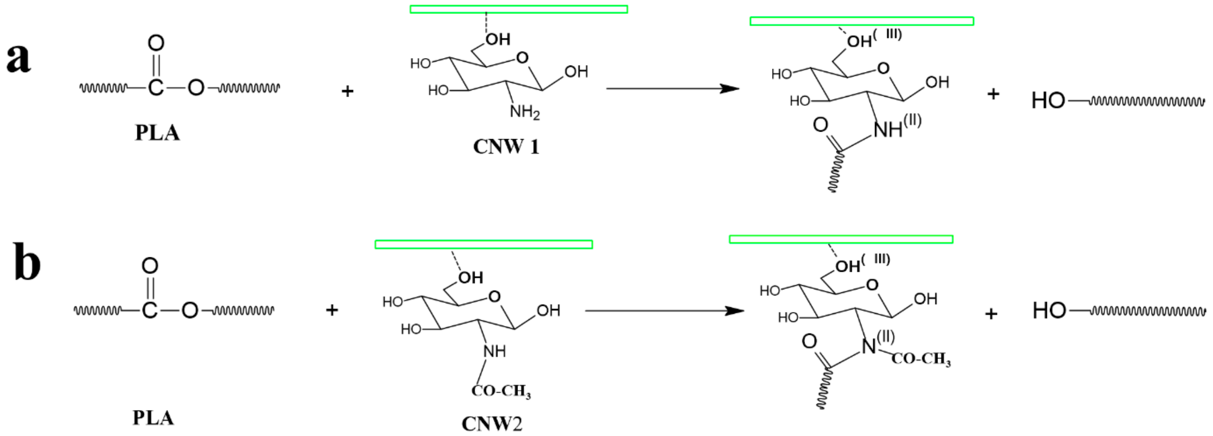

Scheme S1. Aminolysis of polyester chains by a) primary and b) secondary amines on surface of CNW.

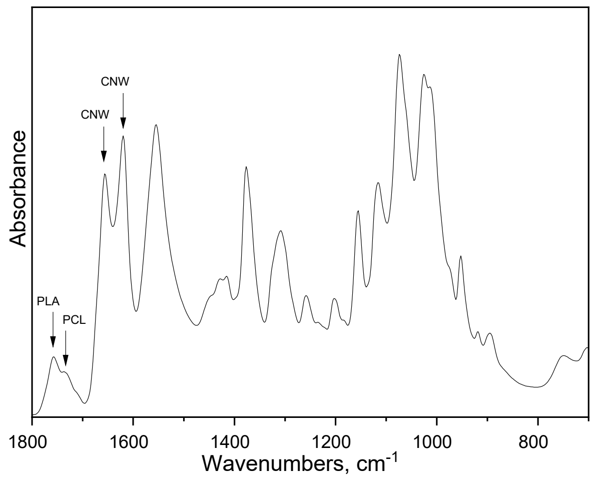

Figure S2. FTIR of CNW extracted from PCL/PLA/CNW nanocomposite using modified filtration process (consisting of replacement of solution over the filter by fresh solvent).

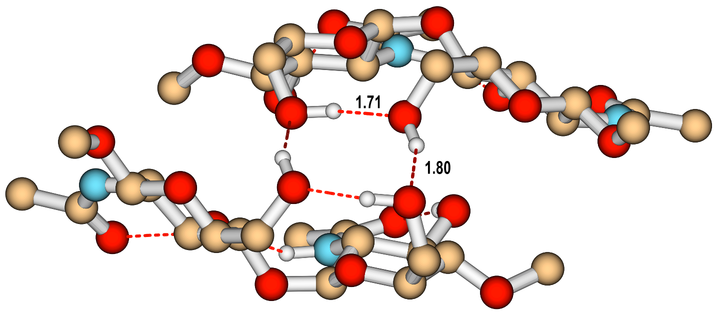

Stabilization energy: 40.6 kcal/mol

(a) DFT model calculations of interactions between two structural units of Chitin.

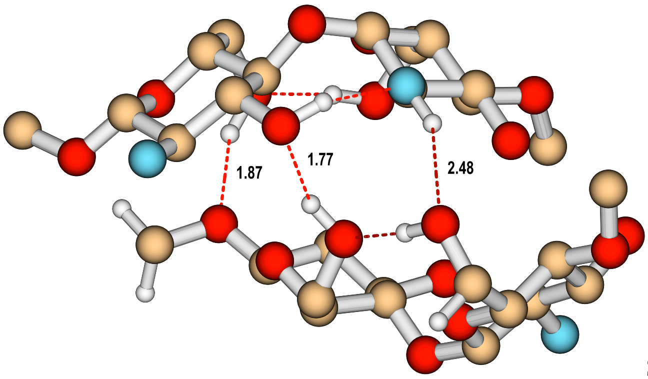

Stabilization energy: 37.0 kcal/mol

(b) DFT model calculations of interactions between two structural units of Chitosan.

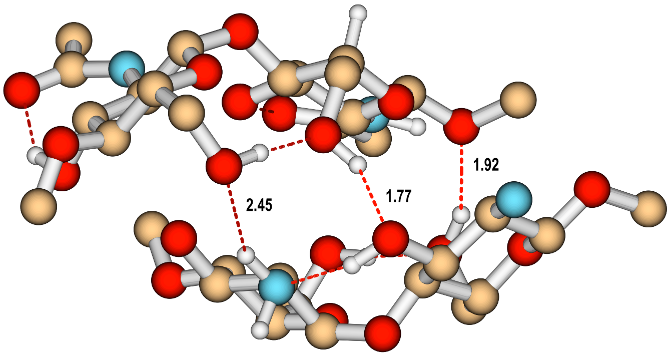

Stabilization energy: 40.8 kcal/mol

(c) DFT model calculations of interactions between the structural units of Chitin and Chitosan.

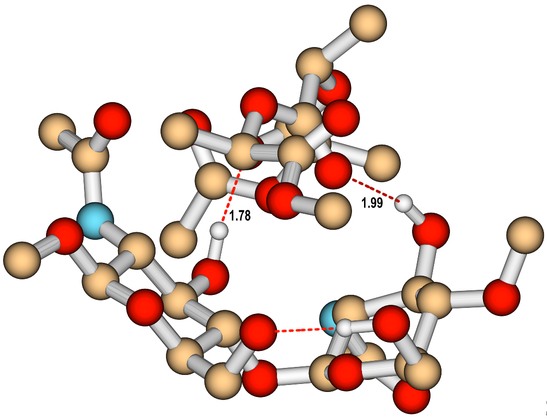

Stabilization energy: 36.8 kcal/mol

(d) DFT model calculations of interactions between the structural units of Chitin and PLA.

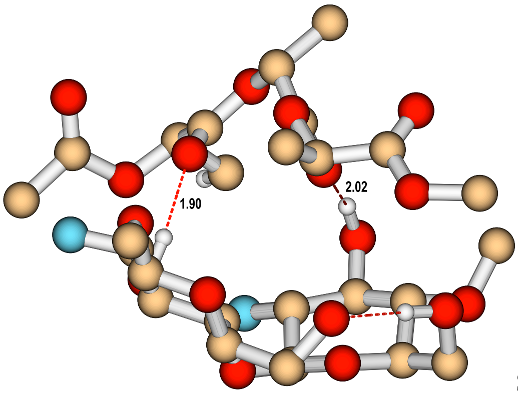

Stabilization energy: 31.1 kcal/mol

(e) DFT model calculations of interactions between the structural units of Chitosan and PLA.

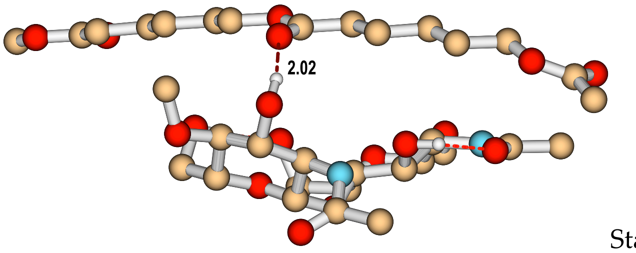

Stabilization energy: 21.1 kcal/mol

(f) DFT model calculations of interactions between the structural units of Chitin and PCL.

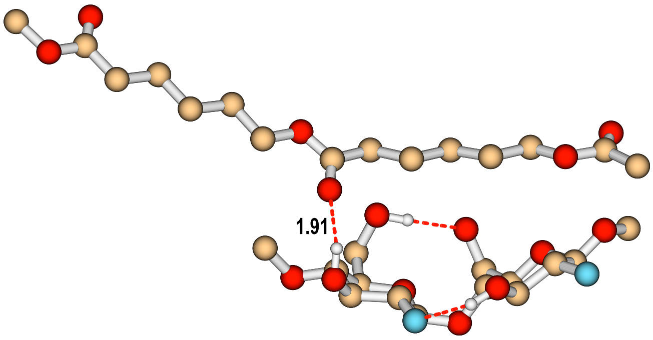

Stabilization energy: 21.7 kcal/mol

(g) DFT model calculations of interactions between the structural units of Chitosan and PCL.

Scheme 2. DFT model calculations of interactions between the structural units of polymer and CNW components. Hydrogen atoms are omitted for clarity.

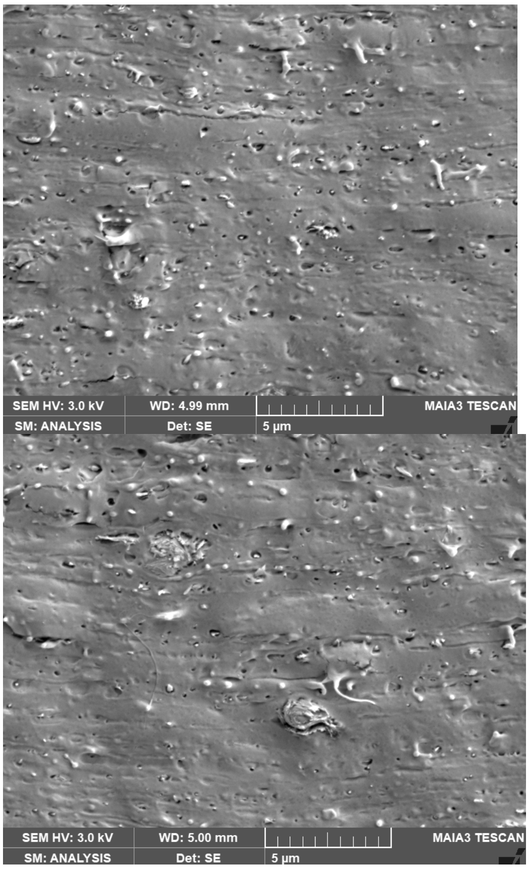

Figure S3. Fracture surface of PCL/PLA/CNW system indicating possible presence of PLA fibres.

Table S2. Modulus of PCL/PLA blend in dependence on structure estimated using basic models E_{PCL}=302 MPa, E_{PLA}=2630MPa.

Structure type	E (MPa)	Model
bicontinuous	1001	Davies [47]
PCL matrix/PLA inclusions	811	Kerner[48]
PCL matrix/PLA fibers (AR10)	1285	Halpin Tsai[49]
PLA matrix/PCL inclusions	1264	Kerner[48]
PLA matrix/PCL fibers (AR10)	1440	Halpin Tsai[49]
Bicontinuous, 10% of PLA fibers (AR10)in PCL In PCL phase	1106	Halpin Tsai [49]+Davies [47]
Bicontinuous, 10% of PLA spheres In PCL phase +10% of PCL spheres In PLA phase	1007	Kerner [48]+Davies [47]
Bicontinuous, 10% increase of PCL modulus phase by CNW (~335 MPa)	1043	Davies [47]
Bicontinuous, 10% increase of PLA modulus phase by CNW (~2840 MPa)	1048	Davies [47]
